# Association between traumatic brain injury (TBI) patterns and mortality: a retrospective case-control study

**DOI:** 10.12688/f1000research.54658.1

**Published:** 2021-08-11

**Authors:** Gilbert Koome, Faith Thuita, Thaddaeus Egondi, Martin Atela

**Affiliations:** 1 School of Public Health, University of Nairobi, Nairobi, 00200, Kenya; 2 Strathmore Institute of Mathematical Sciences, Strathmore University, Nairobi, 00200, Kenya; 3 Peterhouse, University of Cambridge, Cambridge, Cambridge, UK

**Keywords:** Trauma Patterns, Patient Characteristics, pre-hospital Care, Traumatic Brain Injuries, Trauma mortality

## Abstract

**Background**: Low and medium income countries (LMICs) such as Kenya experience nearly three times more cases of traumatic brain injury (TBI) compared to high income countries (HICs). This is primarily exacerbated by weak health systems especially at the pre-hospital care level. Generating local empirical evidence on TBI patterns and its influence on patient mortality outcomes is fundamental in informing the design of trauma-specific emergency medical service (EMS) interventions at the pre-hospital care level. This study determines the influence of TBI patterns and mortality.

**Methods: **This was a case-control study with a sample of 316 TBI patients. Data was abstracted from medical records for the period of January 2017 to March 2019 in three tertiary trauma care facilities in Kenya. Logistic regression was used to assess influence of trauma patterns on TBI mortality, controlling for patient characteristics and other potential confounders.

**Results:** The majority of patients were aged below 40 years (73%) and were male (85%). Road traffic injuries (RTIs) comprised 58% of all forms of trauma. Blunt trauma comprised 71% of the injuries. Trauma mechanism was the only trauma pattern significantly associated with TBI mortality. The risk of dying for patients sustaining RTIs was 2.83 times more likely compared to non-RTI patients [odds ratio (OR) 2.83, 95% confidence interval (CI) 1.62-4.93, p=0.001]. The type of transfer to hospital was also significantly associated with mortality outcome, with a public hospital having a two times higher risk of death compared to a private hospital [OR 2.18 95%CI 1.21-3.94, p<0.009].

**Conclusion: **Trauma mechanism (RTI vs non-RTI) and type of tertiary facility patients are transferred to (public vs private) are key factors influencing TBI mortality burden. Strengthening local EMS trauma response systems targeting RTIs augmented by adequately resourced and equipped public facilities to provide quality lifesaving interventions can reduce the burden of TBIs.

## Abbreviations

AOR, adjusted odds ratio; CI, confidential interval; CNS, central nervous system; EMS, emergency medical services; GCS, Glasgow Coma Scale; LMIC, low- and medium-income country; RTI, road traffic injury; SE, standard error; TBI, traumatic brain injury; TI, traumatic injury.

## Introduction

Trauma is a serious global public health problem. ^1^ Traumatic injuries (TIs) are estimated to account for 10% of all deaths and about 5.8 million deaths annually and at least 6% of Years Lived with Disability (YLD). ^2, 3^ Low and medium-income countries (LMICs) account for about 90% of this global trauma disease burden. ^4^ Globally, traumatic brain injury (TBI) is the leading form of TI burden. ^1^ Currently, about 69 million people suffer from TBI annually, mainly from road traffic injuries (RTIs), violence and falls. ^7, 8^ The estimated economic cost of RTIs in Europe is substantially high with an approximate range of 7500-1,200,00 US dollars. ^5^ Young persons aged less than 40 years are the most affected. ^6^ In the EU, over 1.5 million people are admitted to hospital for TBI annually, with Austria and Germany reporting about eight times more admissions compared to Portugal and Spain. Of these admissions, about 50,000 deaths, 230,000 hospital admissions and 1.1 million discharges are reported. ^7^ One study found EU hospital admissions, adjusted for population, to be three times higher compared to the USA. ^7^ This indicates significant inter-continental TBI burden and trauma system development variations.

LMICs, mainly in Africa, experience about three times more cases of TBIs compared to high income countries (HICs) such as EU and USA. ^8^ This constitutes approximately 80% of the TBI global burden, most cases of which are potentially preventable using quality and effective pre-hospital care emergency medical service (EMS) systems. ^4, 9^ In these countries, TBI remains a growing public health burden concern. There are increasing concerns that EMS at the pre-hospital care level is ineffective and incapable of adequately mitigating increasing number of TBIs requiring critical care interventions. ^10^ The economic cost of TBIs, such as mortality, morbidity and high hospital bills, has serious economic impact at an individual, household and societal level. ^11^ For instance, LMICs, mainly in sub-Saharan Africa, lose about four billion United State dollars (US$) annually due to RTIs, a major cause of TBIs. This is equivalent to 11% of the their gross domestic product (GDP). ^10, 12^ In Kenya, the cost of RTIs is estimated at 14 billion Kenya shillings per year. ^13^

In Kenya and other LMICs, the greatest proportion of TBI mortality and morbidity burden is attributed to poor access to quality emergency care. ^14^ In HICs such as America and Europe, about half of such preventable mortality is said to occur at the pre-hospital care level. ^15^ The proportion and impact is estimated to be three times as high in LMICs. ^8^ The disproportionately high burden and cost of TBI in Africa and other LMICs are exacerbated by weak health systems, especially at the pre-hospital care level, and limited reliable empirical evidence to inform effective response to the growing trauma burden. ^4, 16– 19^

Ideally, TBI patients are expected to receive quality pre-hospital care, also known as “life-saving interventions” from trained and qualified health professionals before pre-hospital transport and/or reaching a specialized trauma care facility, also know as Tertiary Facilities. However, few patients receive this care, with most victims receiving no life-saving interventions due to lack of qualified staff and resourced EMS facilities at this care level. ^14^ Evidence affirms a lack of a well-coordinated and integrated pre-hospital trauma care system in these settings to respond to growing TBI burden. ^20^ Thompson *et al.* ^21^ described Kenya's EMS for pre-hospital trauma care such as TBIs as fragmented, poorly coordinated and ineffective. Weakness in the health system has been linked to, among others, inadequate resources, staff, leadership, lack of training standard, lack of emergency trauma specialists, lack of effective communication systems and ineffective EMS response systems. ^22– 24^ In Kenya and other LMICs, evidence to support the development of local and adaptive life-saving interventions for averting or reducing growing TBI mortality at pre-hospital care level are grossly lacking. ^11, 25^

TBI patterns comprise a complementary component of a responsive trauma/injury assessment and response at the pre-hospital care level. For instance, effective EMS response may require responses matched to the specific trauma source, injury type and its severity level. ^26^ In this study, trauma pattern is defined in three different ways; based on source (RTIs and non-RTI), type of injury (blunt and penetrating) and day of injury (weekday and weekend). Reviews indicate the main cause of TBIs is RTIs, followed by violence and falls. ^10, 22, 27^ TBI burden from RTIs has continued to exert pressure on already weak health systems globally due to increasing motorization and lack of effective EMS response systems. ^28– 31^ For instance, worldwide, about 1.2 million RTIs occurred in 2012, with male persons aged 15-29 years being the most affected. ^32^ In Ghana, about 50% of TIs (TBIs included) are reportedly traffic and falls-related. ^22, 27^ In Kenya, more than 75% of health facilities’ emergency department visits are also due to RTIs. ^12^ In LMICs alone, RTIs are estimated to increase by about 80% in the next decade due to an increase in motorization, low adherence to road traffic regulations and lack of effective EMS at the pre-hospital care level. ^10^

Source of trauma has been associated with increasing mortality burden from all trauma forms - RTIs have been linked to increasing burden of all forms of trauma mortality ^33– 35^ but no similar findings on TBI mortality have been found. Limited evidence available indicates that penetrating trauma or injuries are associated with a significantly longer length of stay in hospital. ^28^ Penetrating trauma has also been found to increase all forms of trauma mortality significantly. ^36, 37^ Blunt trauma has been closely linked to motor vehicle collisions and falls. ^38^ No study was found to examine the relationship between TBI type and mortality in LMICs. Access to prompt and quality care, at both the pre-hospital and in hospital level, can vary substantially by day of injury. Availability of trained staff and health services is higher during weekdays compared to weekends. ^39, 40^ For instance, during weekends, there could be fewer trained providers to promptly respond to EMS needs. Available studies focus mainly on injury admission day - which may be different from injury day considering long pre-hospital delays in Kenya and other LMICs - and are mainly descriptive. ^19, 28, 39^ These studies report higher prevalence of TIs during weekends than on weekdays due to high trauma risk activities such as social events and mobility. According to a South African study by Möller *et al.*, hospital admission day is significantly associated with all forms of trauma mortality. ^36^ However, studies examining the influence of TBI day on mortality could not be found in the literature.

In Kenya and other low resource countries, preventive public health measures such as road safety measures and laws like using motorcycle helmets and observing road traffic regulations, have been instituted to reduce trauma burden and address some of the identified EMS response weaknesses. These measures seem to have failed to substantially reduce mortality and disability arising from TBIs and other TIs. ^17^ Understanding TBI patterns and their influence on mortality can provide important insight on occurrence, presentation, diagnosis and alternative interventions for improving patient survival outcomes. Characterizing these patterns can enable EMS and other health professionals to understand and mitigate presentation of TBIs and possible risk factors for avoidable mortality. ^30, 41^ The evidence can also offer critical insight in designing locally adaptive and effective responses for the pre-hospital trauma care level, yet this evidence is widely lacking in locally published literature. It is with this background that this study aimed to test the hypothesis that the patterns of TBI was not associated with mortality at the pre-hospital care level.

## Methods

### Data abstraction

We conducted a retrospective unmatched case-control study through data abstraction from hospital-based patient records. This study was done in three leading tertiary referral facilities comprising public (Kenyatta National Hospital, KNH) and private (Kikuyu Mission and Mater Misericordiae Hospital) tertiary trauma facilities in Kenya. Data was abstracted from 316 TBI patient medical records (consisting of 158 cases and 158 controls) for the period of January 2017 to March 2019 in the three hospitals. The study sample was calculated using Kelsey’s unmatched case control formula. ^42^ The formula assumptions were as follows, proportion of cases exposed; 0.667, proportion of controls exposed; 0.6 and case-control ratio of 1.0. Cases were patients who had died within 30 days after the trauma while controls were patients who survived for at least 30 days after trauma. At the facility, all TBI patients meeting the inclusion criteria were sampled and listed. Patients with TBIs were identified using International Classification of Diseases (ICD) codes assigned to patient medical records or files. A separate list of cases and controls which met the study inclusion criteria was developed. In situations where more than one control was identified or available, simple random sampling using a random number generator was used to sample one control from the list. Controls for cases were derived from the same facility to mitigate bias associated with difference in quality of care in different facilities.

In the study, patients with TBIs based on ICD diagnosis codes received in the three selected trauma referral hospitals for the period between January 2017 to March 2019 were included. Immediate death on the scene and patients not transferred to hospitals due to minor injuries were excluded from the study. Immediate deaths on injury scene due to severe injuries presents little opportunity for improving care through life-saving interventions while minor injuries such as bruises presents no major mortality risks. 

During sampling, immediate cases/deaths occurring at the injury scene were excluded due to inability to obtain comprehensive data and the minimal opportunity for providing pre-hospital care or life-saving interventions. Further, patients admitted in lower level facilities for more than 24 hours after injury were excluded due to potential of confounding linked to differences in care quality in the two levels of care delivery, that is, lower and tertiary level facilities. Pediatric trauma may require different critical care response compared to adult trauma, hence exclusion of patients aged less than 18 years to avoid response-specific bias. Patient records with missing information of at least 5% of the abstracted data were also not included in the analysis.

### Variable(s) description

Data abstracted included mortality outcomes, that is: whether the patient died or survived; trauma patterns comprising day of injury (Monday/Tuesday/Wednesday/Thursday/Friday/Saturday/Sunday), type of injury (penetrating/blunt) and injury mechanism (RTI/non-RTI such as violence, falls and gunshots); demographic characteristics comprising age (18-29 years/30-39 years/40-49 years/50-59 years/60+ years) and gender (male/female) and vital patient characteristics consisting of Glasgow Coma Scale (GCS) score (severe, moderate and mild), presence of hypoxemia, defined as blood oxygen concentration of less than 90%, (Yes/No), presence of comorbidity (Yes/No), alcohol involvement (Yes/No), patient triage status (not urgent/urgent/emergency) and blood pressure levels (hypertension/elevated/normal). We also collected data on access to pre-hospital care categorized as Yes/No. To improve completeness and accuracy, abstracted data were complemented with other pre-hospital care records including ambulance records, referral notes and trauma registries maintained at the Accident and Emergency Departments. For deceased patients, mortality and death notification reports were used to collate information on death which included cause of death, place of death and the patient demographics comprised of age and gender.

### Statistical analysis

For descriptive analysis, we performed bivariate analysis to assess mortality outcome differences among cases and controls, including both individual and trauma pattern characteristics. We used Pearson’s chi-square test to assess differences in proportions and Student’s t-tests to assess differences in mean patient ages. Logistic regression was used to assess association between mortality and trauma patterns adjusting for other predictor variables. To identify possible confounding effects, existence of a 10% difference between unadjusted and unadjusted regression coefficient was used. ^43, 44^ Statistical significance at 0.05 level between exposure and outcome was also taken into account. ^43^ Using these statistical methods, type of transfer tertiary facility and trauma severity score (GCS) were found to confound TBI mortality outcome, hence they were included in the analysis as control variables. Access to life-saving interventions at the pre-hospital care level may result in lower mortality risks for the different trauma patterns. Access to pre-hospital care was defined as the provision of life-saving interventions by a qualified health professional such as a paramedic, nurse, clinical officer or medical officer. To account for this possible confounding effect in outcome, we included access to pre-hospital care as an important control variable in the model. Based on previous studies and other published literature, we also selected patient and injury characteristics that were clinically and substantively (statistically significant at bivariate analysis level) relevant to be included in the adjusted logistic regression model. In this paper, we report the adjusted odds ratio (AOR) for mortality, after controlling for age, gender, trauma severity, presence of hypoxemia, presence of comorbidity, pre-hospital time, type of transfer tertiary facility and access to pre-hospital care. Due to small sample size in some of the age and injury day variable categories, some of the categories were combined in the regression model - age was re-constituted to three categories (18-29 years/30-39 years/40+ years and injury day to two categories (weekday and weekend). Abstracted medical data was analyzed using IBM SPSS statistics software, version 26. A p-value of less than 0.05 was considered statistically significant.

### Ethics statement

Since the study involved de-identified retrospective data abstracted from many patient records - some of whom were deceased - it was difficult to reach and contact all the respondents and obtain informed consent, particularly, the deceased. A waiver of consent for data abstraction was granted by the Kenyatta National Hospital- and University of Nairobi Ethics and Research Committee (KNH-UoN/ERC/FORM/IC05). A research permit was obtained from the National Commission for Science, Technology and Innovation (NACOSTI/P/19/9613/31326). Institutional ethical clearance was also obtained from all the three hospital ethical boards prior to data collection. There is no identification or individual details presented in this article or data thereof. This is in line with the waiver for consent obtained during ethical approval of this study.

## Results

### Descriptive summary

A descriptive summary of patient characteristics and mortality outcomes is shown in Table 1. The mean age of patients was 34.5 years and there was no significant difference between cases and controls. Eighty-five percent of the patients were males. The distribution of gender was similar in both cases and controls (82% versus 88%). The distribution of blood pressure levels (hypertension, elevated and normal) was the same among cases compared to controls. The number of severely injured patients was significantly higher among cases compared to controls (65% versus 34%). Similarly, the number of patients triaged as non-urgent was significantly higher among cases than controls (30% versus 22%). However, the number of patients triaged as emergency cases was significantly higher in controls compared to cases (47% versus 41%).

**Table 1.** Patient characteristics summary by mortality outcome (cases and controls). Statistical significance (Probability(P) values) is shown in asterisks. Number of cases are 158, Controls are 158 and total population are 316 persons. In the table, + means “and above”, “<” means less than and “>” means more than. Parenthesis (-) shows range of values while % means percentage.

Variable		Mortality outcome			
		Controls (N=158; %)	Cases (N=158; %)	Total (n=316; %)	P-value
Age (mean)		33.89	35.11	34.5	0.552
Age categories	18-29 years	71(45)	64(41)	135(43)	0.876
	30-39 years	46(29)	49(31)	95(30)	
	40-49 years	17(11)	21(13)	38(12)	
	50-59 years	14(9)	12(8)	26(8)	
	60+ years	10(6)	12(8)	22(7)	
Gender	Male	139(88)	129(82)	268(85)	0.158
	Female	19(12)	29(18)	48(15)	
Blood pressure	Hypertension	63(40)	72(46)	135(43)	0.117
	Elevated	36(23)	24(15)	60(19)	
	Normal	55(55)	66(42)	121(38)	
Trauma severity (GCS score)	Severe (GCS<9)	54(34)	100(65)	154(49)	0.001 ***
	Moderate (GCS 9-12)	37(23)	29(18)	66(21)	
	Mild (GCS 13-15)	67(42)	29(18)	96(30)	
Triage status	Not urgent Urgent Emergency	34(22)	47(30)	81(26)	0.229
		50(32)	47(30)	97(31)	
		74(47)	64(41)	138(44)	
Hypoxemia	Yes	39(25)	60(38)	99(31)	0.001 ***
	No	119(75)	98(62)	217(69)	
Comorbidity	Yes	57(36)	66(42)	123(39)	0.356
	No	101(64)	92(58)	193(61)	
Alcohol use	Yes	35(22)	52(33)	87(28)	0.044 *
	No	123(78)	106(67)	229(72)	
Pre-hospital time	<3 hours	101(64)	79(50)	180(57)	0.027 *
	3-6 hours	24(15)	26(16)	50(16)	
	6+ hours	33(21)	53(34)	86(86)	
Transfer facility	Public	67(42)	100(63)	167(53)	0.001 ***
	Private	91(58)	58(37)	159(47)	

* p≤0.05.

*** p≤0.001.

GCS, Glasgow Coma Scale.

Source: Author.

Hypoxemia was present in 31% of the patients. The number of hypoxemic patients was significantly higher in cases compared to controls (25% versus 38%). Turning to comorbidity, 39% of the patients were diagnosed with comorbidity. The distribution of comorbidity was similar among cases and controls (42% versus 36%). The pattern was different among 28% of the patients who had a history of alcohol use. The distribution of patients with a history of alcohol use was significantly higher among cases compared to controls (33% versus 22%). In respect to pre-hospital time, 57% of patients arrived at the tertiary hospital less than three hours after injury. Distribution of pre-hospital time was significantly different in both cases and controls. There were more patients arriving at the tertiary hospital in less than three hours among controls compared to cases (64% versus 50%). Injured patients are transferred from scenes to different tertiary trauma care hospitals for specialized care and management;53% of patients were transferred to a public tertiary hospital. The number of patients transferred to a public facility was significantly higher among cases compared to controls (63% versus 42%). Around half (56%) of patients received pre-hospital care. The number of patients who accessed pre-hospital care was significantly higher among controls compared to cases (63% versus 49%).

### Trauma patterns

We examined three types of trauma patterns; trauma mechanisms (RTI and non-RTI causes), type of injury (blunt and penetrating) and injury day (weekday and weekend). A descriptive summary of trauma patterns by mortality outcomes is shown in Table 2.

**Table 2.** Descriptive summary of trauma patterns by mortality outcomes. Statistical significance (Probability(P) values) is shown in asterisks. Number of cases are 158, Controls are 158 and total population are 316 persons. In the table, Parenthesis (-) shows range of values while % means percentage.

Variable		Mortality outcomes		Total (n=316; %)	P-value
		Controls (N=158; %)	Cases (N=158; %)		
Trauma mechanism	RTIs	78(49)	106(67)	184(58)	0.001 ***
	Non-RTIs	80(51)	52(33)	132(42)	
Type of trauma	Blunt injury	103(65)	122(77)	225(71)	0.025 *
	Penetrating injury	55(35)	36(23)	91(29)	
Day of injury	Monday	30(19)	18(11)	48(15)	0.286
	Tuesday	18(11)	26(16)	44(14)	

* p≤0.05.

*** p≤0.001.

RTI, road traffic injury.

Source: Author.

*Trauma mechanisms*

**Figure 1.**
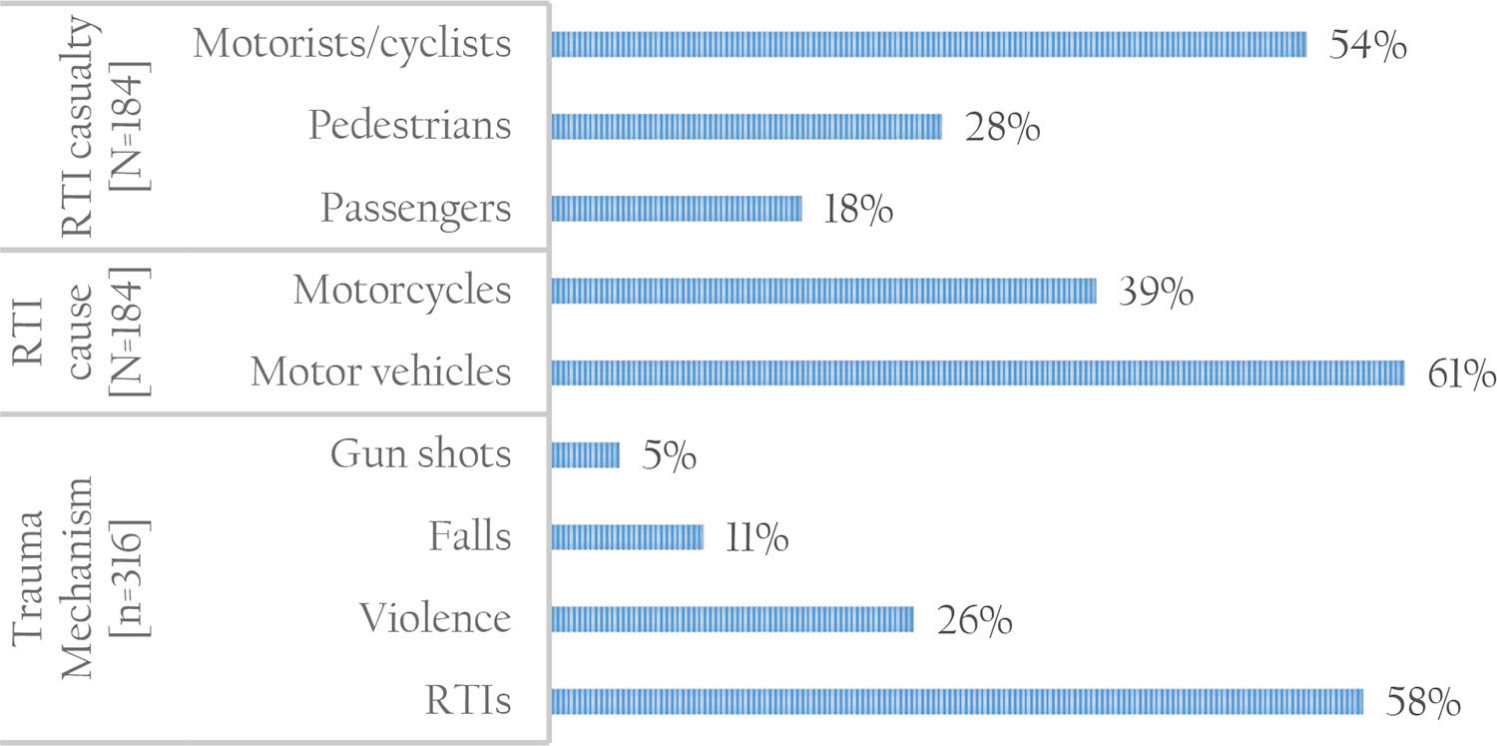
Type of trauma mechanisms and casualties RTI, road traffic injury. Source: Author.

RTIs were the main cause of TBIs (58%) compared to non-RTI causes (42%) which consisted of falls, violence and gunshots ( Figure 1). RTIs and gunshots were the main source of trauma among patients aged 18-29 years, while violence was main source of trauma among patients aged 18-39 years. Motor vehicles (61%) were the main cause of RTIs compared to motorcycles. Trauma caused by gunshots, falls and violence was mainly reported in public places (54%), followed by home (27%) and workplace (18%). TBIs due to RTIs were significantly higher among cases compared to controls (67% versus 49%).

*Type of trauma or injury*

**Figure 2.**
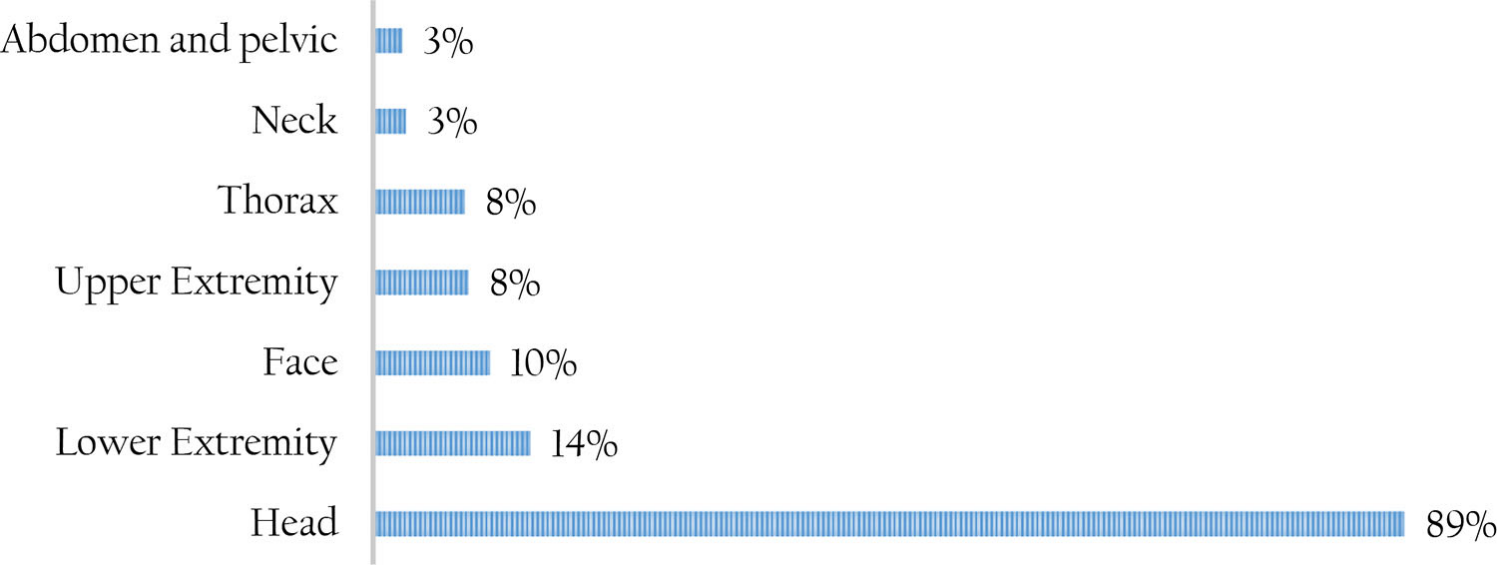
Part of the body most commonly injured among the study patients.

Source: Author.

Blunt trauma (71%) was the main form of injury across all forms of trauma. Blunt trauma was mainly caused by RTIs and falls, while penetrating trauma was mainly caused by gunshots and violence-inflicted injuries. The most commonly injured body part was the head (89%) followed by lower extremities as shown in Figure 2. Record review indicated that concussions and contusions were the main form of head injuries attributed to TBIs. Skull fractures and scalp wounds were also frequently reported. Distribution of blunt trauma was significantly higher among cases compared to controls (77% versus 65%).

*Injury day*

Cumulatively, 72% of injuries were recorded during weekdays, while 28% were recorded during weekends. The weekday has five days, Monday to Friday, while the weekend has only two days, Saturday and Sunday. The number of injuries was slightly higher on Friday compared to other weekdays. However, distribution of injuries was the same across all weekdays.

### Logistic regression

We performed logistic regression analysis (53) to examine association between trauma patterns and TBI mortality. Logistic regression results are shown in Table 3. Trauma mechanism was found to be significantly associated with TBI mortality both independently and after adjusting for other variables. RTI patients were 2.83 times more at risk of dying compared to non-RTI patients. Type of injury was found to be significantly associated with TBI mortality independently but this association became insignificant after adjusting for other variables. The risk of dying from blunt trauma was found to be 1.22 times higher compared to penetrating trauma. Further, injury day was not found to be significantly associated with TBI mortality both independently and after adjusting for other variables but the risk of TBI mortality was found to be 0.69 times lower in weekday injuries compared to weekend injuries.

**Table 3.** Logistic regression model of trauma patterns and mortality. Statistical significance (Probability(P) values) is shown in asterisks. Number of cases are 158, Controls are 158 and total population are 316 persons. In the table, + mean “and above”, “<” means less than & “>” means more than. Parenthesis (-) shows range of values while % means percentage.

Variable	Unadjusted model		Adjusted model	
	OR (95% CI)	P-value	AOR (95% CI)	P-value
Road traffic injury (RTI)	2.09(1.33-3.30)	0.002 **	2.83(1.62-4.93)	0.001 ***
Blunt trauma	1.22(1.03-1.44)	0.019 *	1.21(1.00-1.46)	0.053
Weekday trauma	0.97(0.59-1.58)	0.900	0.69(0.38-1.24)	0.212
Age categories (ref: 40+ years)		0.725		0.798
18-29 years	0.82(0.48-1.41)	0.476	0.92(0.48-1.78)	0.808
30-39 years	0.97(0.54-1.74)	0.920	1.14(0.56-2.30)	0.715
Female gender	1.64(0.88-3.08)	0.119	2.76(1.29-5.92)	0.009 **
Trauma severity (ref: Severe GCS<9)		0.001 ***		0.001 ***
Severe (GCS<9)	4.28(2.48-7.39)	0.001 ***	3.42(1.84-6.36)	0.001 ***
Moderate (GCS 9-12)	1.81(0.94-3.48)	0.075	1.54(0.73-3.24)	0.258
Presence of hypoxemia	4.36(2.58-7.36)	0.001 ***	4.75(2.60-8.68)	0.001 ***
Presence of comorbidity	1.27(0.81-2.00)	0.299	1.82(1.03-3.22)	0.040 *
Alcohol use	1.72(1.05-2.85)	0.033 *	2.57(1.40-4.73)	0.002 **
Pre-hospital time (ref: 6+ hours)		0.026 *		0.639
<3 hours	0.49(0.29-0.82)	0.007 **	0.39(0.02-8.39)	0.545
3-6 hours	0.67(0.33-1.36)	0.274	0.71(0.30-1.67)	0.432
Public tertiary facility (KNH)	2.34(1.49-3.68)	0.001 ***	2.18(1.21-3.94)	0.009 **
Access to pre-hospital care	0.58(0.37-0.91)	0.018 *	0.58(0.03-12.37)	0.728

* p≤0.05.

** p≤0.01.

*** p≤0.001.

AOR, adjusted odds ratio; CI, confidence interval; GCS, Glasgow Coma Scale; KNH, Kenyatta National Hospital; OR, odds ratio.

At the individual level, patient age was not found to be significantly associated with TBI mortality even after adjusting for other variables but the risk of dying was found to be 0.92 times lower among younger patients aged 18-29 years compared to older patient aged 40+ years. Female gender was found to be significantly associated with TBI mortality after adjusting for other variables but not independently. A female patient was 1.64 times more at risk of dying compared to a male patient. Trauma severity was also found to be significantly associated with TBI mortality both independently and after adjusting for other variables. The risk of dying from a severe trauma (GCS<9) was 3.42 times more likely compared to mild or minor trauma (GCS 13-15). After adjusting for other variables, a hypoxemic patient was found to be significantly associated with a 4.75 times higher risk of dying compared to a non-hypoxemic patient.

Presence of comorbidity was significantly associated with TBI mortality after adjusting for other variables but not independently. The risk of dying was 1.82 times more likely in comorbid patients compared to non-comorbid patients. Alcohol use was also significantly associated with TBI mortality both independently and after adjusting for other variables. Patients with a history of alcohol use were 2.57 times more at risk of dying compared to those with no alcohol use history. Pre-hospital time was not found to be significantly associated with TBI mortality after adjusting for other study variables but shorter pre-hospital transport time seemed to reduce the risk of dying. In respect to type of transfer tertiary facility, type of facility was found to be significantly associated with TBI mortality both independently and after adjusting for other variables. Transfer of patients to a public facility was associated with a 2.18 times higher times risk of dying compared to a private facility. Access to pre-hospital care was not found to be significantly associated with TBI mortality after adjusting for other variables but was found to lower TBI mortality risk by 0.58.

## Discussion

This study sought to assess the association between trauma patterns and TBI mortality. We found one trauma mechanism in particular, RTIs, to be associated with a higher risk of TBI mortality. Trauma mechanism, mainly TRIs, has been previously studied and associated with all forms of trauma mortality. ^33– 35^ However, these studies did not adjust for access to pre-hospital care and type of trauma care facility, which are shown to be important confounders of mortality outcome. Globally, and particularly in sub-Saharan Africa, RTI is a major public health problem contributing to a high burden of post-trauma mortality and morbidity. ^28, 33, 34, 36^ More than 50% of trauma is RTI-related. ^22^ In Africa and other LMICs such as Ghana, India, Kenya and Uganda, the negative impact of an increase in motorcycle injuries is an increasing public health problem reflected by huge cases of RTI-related fatalities and morbidities. ^22, 28, 33, 34^ Kenya has seen an unprecedented increase in motorcycle motorization, which has exponentially increased trauma burden such as mortality and disability. ^33^ In some LMICs, injury burden from motorcycles has exceeded those from other motorized vehicles. ^28, 33^ This has been worsened by lack of EMS care capacity at the pre-hospital care level to respond to increasing demand for quality care in settings outside of hospital. ^45^

Pedestrians, unrestrained passengers and motorists are the most commonly injured patients due to high exposure risks when crossing roads and walking along major roads. In addition to non-adherence to road traffic rules among motorists, poor road infrastructure contributes to increased risk of RTIs. ^16, 22^ For instance, many major roads have limited provisions for safe walkways and cycling paths, further exposing road users to avoidable injuries. Road safety and awareness campaigns have not been adequately successful in injury and mortality risk reduction. ^46, 47^ Complementing these efforts with effective on-scene EMS responses can provide possible survival and health outcome benefits. This requires continued mapping and review of trauma pattern profiles as well as their effect on mortality in both rural and urban areas to inform trauma-specific responses. Consistent with other studies, ^28^ the head was found to be the most injured body part. This is expected because our study population are TBI patients. Chalya *et al.* ^28^ reported head injuries are the leading cause of deaths and disability in TBIs. Head injuries are associated with an increase in odds of death of around 1.5 times ^48^ compared to non-head injuries. Similar to this study, lower extremities were also found to be frequently injured. The limbs sustain wounds, abrasions laceration, amputations or fractures which constitute significant morbidity risks such as disability as well as deaths due to uncontrolled hemorrhage. Serious head injuries present with life-threatening outcomes such as intercranial hemorrhage, which require timely life-saving interventions. Brain contusion and concussions were frequent form of injuries attributed to blunt injuries. These brain injuries can pose significant mortality risks if not managed effectively and promptly in line with the "Golden Hour" concept. ^33, 45^ This concept argues that life-saving interventions are be provided within one hour after injury for high efficacy while preventing irreversible pharmacological changes associated with higher mortality risks. Internal head and brain injuries are easily missed (missed injuries) due to poor diagnosing capability and lack of trained staff at the pre-hospital care level. Gaps in pre-hospital life-saving responses significantly increase the risk of preventable mortality. ^33, 45^ Quality pre-hospital trauma responses matching skill and resource needs are lacking in these settings, hence a higher case load of avoidable fatalities and disabilities. This burden can be averted using effective and timely out of hospital settings EMS responses targeting this type of trauma.

Blunt trauma is frequently reported in RTIs ^37, 38^ while penetrating trauma is frequent in gunshots, stabs and other invasive trauma. Type of injury (blunt or penetrating) was not a predictor of TBI mortality after adjusting for other variables and confounders. This is inconsistent with other studies which showed penetrating trauma to increase mortality significantly. ^36, 37^ However, unlike our study, in these studies, which included other types of trauma such as central nervous system (CNS) injuries and amputations, penetrating trauma was most frequently reported. This variation in trauma mechanism and pattern may partially explain the difference in results. Blunt trauma was associated with increased odds of mortality, although this association was not significant. This is attributed to the role of RTI which is the main source of blunt injuries.

We further investigated the effect of injury day on trauma mortality. We found distribution of injury to be similar across weekdays. There was also no significant difference in TBI outcomes when weekend and weekday injuries were compared. While the study doesn’t provide sufficient evidence to refute the existing argument that TI (morbidity) is a predominantly a weekend problem due to high number of social events and mobility, ^19, 28, 39^ it suggests that TBI morbidity may not be predominantly a weekend problem as expected. We found no published study examining the influence of injury day on trauma mortality outcomes. One study by Möller *et al.* examined a different but related aspect, the influence of hospital admission day on all forms of trauma mortality. ^36^ The study showed high trauma case admissions during weekends, especially between midnight and six o’clock in the morning compared to weekdays. ^36^ In the study, hospital admission day was significantly associated with mortality in which more deaths were noticeable on Tuesdays and Fridays. However, due to the small sample size in which the observed cell frequencies for these days was less than five, generalization of the evidence was low.

Further, a descriptive study from Tanzania found higher rates of injuries during the day but did not examine the influence of day of injury dynamics on mortality. ^28^ We note that during the day, injury exposure is high due to higher mobility due to travel to workplaces, engagement in economic activities and other social events. Our study did not examine the difference between day and night injuries due to a lack of data on this injury characteristic. A lack of robust and comprehensive trauma registries across LMIC countries that capture injury day and other injury data is the main reasons for this study gap. As a result, most authors use trauma admission time, which is easily found in patient records, as proxy indicator of injury day. In LMICs, pre-hospital delays can be as high as seven days due to missed injuries and under-triaging and hence are a possible reason for the difference in mortality outcome between injury day and admission day after trauma.

We also included type of transfer facility in the adjusted predictor model. To our knowledge, this is the first published study to control for type of tertiary facility as an important contextual and control factor that can significantly alter trauma mortality outcomes. We found type of tertiary transfer facility to be significantly associated with TBI mortality, both independently and after adjustment of other variables. In this study, risk of dying was significantly higher among patients transferred to a public tertiary facility compared to a private facility. Access to pre-hospital care (from trained EMS providers) and differences in care among public and private transfer facilities can alter post-trauma outcomes due to differences in quality of life-saving and trauma care interventions provided as part of care continuity. Patient transfer to well-equipped facilities can have substantive benefits for survival outcomes. In LMICs, private facilities are known to be better equipped and staffed than public facilities, hence creating variations in care quality. For instance, at minimum, a well-equipped trauma care facility should provide computerized tomography (CT) scanning, hemorrhage control, provision of IVs, neurosurgical care, Intensive Care Unit, intracranial monitoring and treatment among others as part of critical care. ^49^ Availability of these facilities, resources and equipment may vary substantively across public and private facilities.

In Kenya and other LMICs, most public referral facilities have limited resources to attend to a high number of patients linked to poor leadership and underfunding, hence possible suboptimal care. ^9, 17^ There are also care delays due to over-triaging and ineffective governance systems to support timely and optimal trauma care compared to public facilities. ^14, 26^ In public facilities, there are limited theatres and experts to serve a high number of casualties especially in cases of mass casualty. ^49^ Qualified trauma specialists are also frequently away in public practice, leading to delays in life-saving care. In private tertiary facilities, trauma care is promptly provided due to manageable numbers of TBI patients seeking care. ^50^ The facilities are also well equipped and resourced to provide specialized care with readily available specialists. This allows maximal care, leading to improved outcomes compared to public facilities. However, high medical fees in private facilities, combined with lack of comprehensive health insurance among the poor and most vulnerable result in limited access to private facility trauma care services. ^19, 20^ Addressing gaps in capacity of public facilities has been identified as an important intervention to reduce avoidable pre-hospital mortality and morbidity burden most prevalent among the less well-off. ^6, 10, 51^

## Conclusion

Trauma mechanism (RTI and non-RTI) and type of tertiary facility patients are transferred to (public vs private) are key factors influencing TBI mortality burden. Strengthening local trauma emergency care responses targeting RTIs and equipping public health facilities to provide quality and timely critical trauma care is indicated. Addressing post-trauma care access barriers in private tertiary facilities such as through comprehensive social insurance for the poorest and vulnerable can also significantly increase timely access to quality life-saving interventions and reduce TBI mortality burden from RTIs.

## Data availability

### Underlying data

Harvard Dataverse: Association between Traumatic Brain Injury (TBI) patterns and mortality. https://doi.org/10.7910/DVN/TF4LXE ^52^

This project contains the following underlying data:

• Data Gilbert_SPSS Data-Jo- 1.tab

This project also contains the following extended data:

• Medical Records Review Checklist.docx

### Extended data

Harvard Dataverse: Replication Data for: Association between Traumatic Brain Injury (TBI) patterns and mortality. https://doi.org/10.7910/DVN/TK8BXF

This project contains the following extended data:

• Data Gilbert_SPSS Data-Jo- Hav.tab

Data are available under the terms of the Creative Commons Zero “No rights reserved” data waiver (CC0 1.0 Public domain dedication).
