# Supplementary material for: Association between traumatic brain injury (TBI) patterns and mortality: a retrospective case-control study
Source: F1000Res. 2022 Jan 31;10:795. Originally published 2021 Aug 11. [Version 2] doi: 10.12688/f1000research.54658.2 (PMC8829093; doi:10.12688/f1000research.54658.2)
Supplement: Peer review history

# Peer review: Reviewer response for version 2

**DOI:** 10.5256/f1000research.120555.r121667

**Published:** 2022-02-09

**Authors:** Amit Agrawal

**Affiliations:** 1 Department of Neurosurgery, All India Institute of Medical Sciences, Bhopal, Bhopal, Madhya Pradesh, India

The authors have made appropriate changes.

Is the work clearly and accurately presented and does it cite the current literature?

Partly

If applicable, is the statistical analysis and its interpretation appropriate?

Partly

Are all the source data underlying the results available to ensure full reproducibility?

Partly

Is the study design appropriate and is the work technically sound?

Partly

Are the conclusions drawn adequately supported by the results?

Partly

Are sufficient details of methods and analysis provided to allow replication by others?

Partly

Reviewer Expertise:

Neurosurgery

I confirm that I have read this submission and believe that I have an appropriate level of expertise to confirm that it is of an acceptable scientific standard.

# Peer review: Reviewer response for version 1

**DOI:** 10.5256/f1000research.58163.r93345

**Published:** 2022-01-19

**Authors:** Peter Kithuka

**Affiliations:** 1 Department of Health management and Health Informatics, Kenyatta University, Nairobi, Kenya

This article provides answers to the hitherto unclear research gaps with respect to the association between traumatic brain injury patterns and mortality. This paper addresses important public health issues which have attracted limited attention in Africa over time. The authors' methodology is scientifically sound in which the findings are well anchored. The conclusions of this study will go a long way in informing important policy deliberation and further research into this area. Except for a few issues raised that need to be rephrased for clarity, the study has a robust and sound scientific base on this topic. Specifically, check on:

- **Background:** The content is very relevant and logically present. However, some literature, well captured in the discussion can be cut down.
- **Statistical Analysis:** Revise the second sentence in this section to read: "we used Pearson's chi-square test to determine the association between trauma pattern and mortality outcomes".
  The author can also provide more details on the control group used in this study.
- **Results:** Rephrase the first sentence in this section to read: “A descriptive summary of study population characteristics versus mortality outcomes is shown in Table 1."
- **Discussion:** No comments – the discussion section is well anchored on the findings presented.
- **Conclusion:** The conclusion is fairly supported by study results or data. However, the author can consider paraphrasing the first sentence to read: "This study aimed to assess the association between trauma patterns and TBI mortality."
  The conclusion should also be reviewed to focus on trauma patterns (RTI and other patterns studied) rather than the type of facility a patient is transferred to; the type of tertiary facility is presented and incorporated as a control variable in line with the statistical analysis used.

Is the work clearly and accurately presented and does it cite the current literature?

Yes

If applicable, is the statistical analysis and its interpretation appropriate?

Yes

Are all the source data underlying the results available to ensure full reproducibility?

Yes

Is the study design appropriate and is the work technically sound?

Yes

Are the conclusions drawn adequately supported by the results?

Yes

Are sufficient details of methods and analysis provided to allow replication by others?

Yes

Reviewer Expertise:

Health Systems Management

I confirm that I have read this submission and believe that I have an appropriate level of expertise to confirm that it is of an acceptable scientific standard.

# Response

**Published:** 2022-01-27

**Authors:** Gilbert Gilbert

**Affiliations:** University of Nairobi, Kenya

The content is very relevant and logically present. However, some literature, well captured in the discussion can be cut down.

*We deleted literature on page 4 which presented findings on the relationship between trauma patterns and mortality outcomes and trauma statistics from high-income countries. Most of this literature was already incorporated into the discussion. This was done to make the introduction section short and concise.*

 The author can also provide more details on the control group used in this study.

*Additional details on the control group were added.*

 Revise the second sentence in this section to read: "we used Pearson's chi-square test to determine the association between trauma pattern and mortality outcomes"

*Revision is done to read as follows, "We used Pearson's chi-square test to determine the association between trauma pattern".*

 Rephrase the first sentence in this section to read: “A descriptive summary of study population characteristics versus mortality outcomes is shown in Table 1."

*The sentence was reviewed to read, "A descriptive summary of study population characteristics and mortality distribution is shown in Table 1".*

 The conclusion should also be reviewed to focus on trauma patterns (RTI and other patterns studied) rather than the type of facility a patient is transferred to; the type of tertiary facility is presented and incorporated as a control variable in line with the statistical analysis used.

*In the conclusion, deduction on the effect of type of tertiary facility transfer on mortality outcomes was removed to retain the emphasis on the association between trauma patterns and TBI mortality.*

# Peer review: Reviewer response for version 1

**DOI:** 10.5256/f1000research.58163.r94017

**Published:** 2021-10-25

**Authors:** Amit Agrawal

**Affiliations:** 1 Department of Neurosurgery, All India Institute of Medical Sciences, Bhopal, Bhopal, Madhya Pradesh, India

The topic of the current article is pertinent and is of public health importance.

- The introduction is too long and can be shortened to include relevant details.
- It will be interesting to provide the total number of cases of TBI presented to the hospital and how the unmatched sample was drawn (equal number of cases versus controls).
- It will be helpful if the details of the control group can be elaborated (healthy versus others).
- Please provide further details of how ICD codes were assigned or selected as it will help in future studies.
- Please provide further details and numbers of excluded cases.
- It will be interesting to have the details of isolated TBI versus TBI and associated injuries and their outcomes.
- Please elaborate in Table 1 GCS if it is for TBI severity not for Trauma Severity; accordingly the text can be updated.
- In reference to the title of Table 1: "Patient characteristics summary by mortality outcome (cases and controls)" - is it mortality outcome or is it a table of characteristics of the study population with subgroup distribution? Similarly, Table 2, "Descriptive summary of trauma patterns by mortality outcomes".
- In other tables when the objective of the study is to include patients with TBI, the distribution of injuries cannot be generalized, rather it will be a distribution of associated injuries in patients with TBI. Accordingly, the language needs to be modified and sentences may need to be re-phrased.
- Once these are potentially incorporated it will be great if the discussion can be modified and updated as well.
- Please support the conclusion by the study findings and it should reflect the objectives of the study.
- Few references are incomplete and need to be updated.
- It is obvious that retrospective design shall have inherent limitations, the statistical methods have text that can better be shifted to the methods section particularly “Access to life-saving…”.
- Although the study does not provide new information, however, it can be a good contribution to understanding regional (i.e. Kenya in the present case) patterns and burden of traumatic brain injury-related morbidity and mortality.

Is the work clearly and accurately presented and does it cite the current literature?

Partly

If applicable, is the statistical analysis and its interpretation appropriate?

Partly

Are all the source data underlying the results available to ensure full reproducibility?

Partly

Is the study design appropriate and is the work technically sound?

Partly

Are the conclusions drawn adequately supported by the results?

Partly

Are sufficient details of methods and analysis provided to allow replication by others?

Partly

Reviewer Expertise:

Neurosurgery

I confirm that I have read this submission and believe that I have an appropriate level of expertise to confirm that it is of an acceptable scientific standard, however I have significant reservations, as outlined above.

# Response

**Published:** 2022-01-27

**Authors:** Gilbert Gilbert

**Affiliations:** University of Nairobi, Kenya

The introduction is too long and can be shortened to include relevant details  

*We deleted literature on page 4 which presented findings on the relationship between trauma patterns and mortality outcomes and trauma statistics from high-income countries. Most of this literature was already incorporated into the discussion. This was done to make the introduction section short and concise.*

 It will be interesting to provide the total number of cases of TBI presented to the hospital and how the unmatched sample was drawn (equal number of cases versus controls).

*We included the number of TBI cases and controls presented to the facilities in the study period to provide more details on the sampling frame.*

 Please provide further details of how ICD codes were assigned or selected as it will help in future studies

*This was added. The codes are assigned based on the written medical notes (documentation) on patient examination and diagnosis by the clinical care providers.*

 It will be interesting to have the details of isolated TBI versus TBI and associated injuries and their outcomes.

*This analytic request was not at the center of this study – is outside our scope – but details of the sampled injuries are listed in Table 3.*

 It will be helpful if the details of the control group can be elaborated (healthy versus others)  

*Additional details on the control group were added.*

 Please provide further details and numbers of excluded cases.

*Details and the number of excluded cases have been provided.*

 In reference to the title of Table 1: "Patient characteristics summary by mortality outcome (cases and controls)" - is it mortality outcome or is it a table of characteristics of the study population with subgroup distribution? Similarly, Table 2, "Descriptive summary of trauma patterns by mortality outcomes".

 In other tables when the objective of the study is to include patients with TBI, the distribution of injuries cannot be generalized, rather it will be a distribution of associated injuries in patients with TBI. Accordingly, the language needs to be modified and sentences may need to be re-phrased.

*The title of Table 1 was revised to indicate the data presented was on study population characteristics versus mortality distribution rather than patient characteristics and mortality outcomes.*

 Please elaborate in Table 1 GCS if it is for TBI severity not for Trauma Severity; accordingly the text can be updated.

*The use of GCS scores to refer to trauma severity was replaced with TBI severity in the entire document.  * 

 Once these are potentially incorporated it will be great if the discussion can be modified and updated as well.

*The discussion was updated with suggested details including change of trauma severity to TBI severity and use of mortality distribution instead of mortality outcomes.*

 Please support the conclusion by the study findings and it should reflect the objectives of the study.

*In the conclusion, deduction on trauma patterns in which deductions on the effect of type of tertiary facility transfer on mortality outcomes was removed to retain an emphasis on association between trauma patterns and TBI mortality.*
